# The impact of headache specialist density and the introduction of gepants and lasmitidan on prescriptions for acute migraine treatments: a regression and interrupted time series analysis

**DOI:** 10.3389/fneur.2025.1530499

**Published:** 2025-02-07

**Authors:** Leon S. Moskatel, Rebecca Y. Linfield, Niushen Zhang

**Affiliations:** ^1^Department of Neurology, Stanford University, Palo Alto, CA, United States; ^2^Department of Infectious Diseases, Stanford University, Palo Alto, CA, United States

**Keywords:** headache specialist, triptans, gepant, lasmiditan, physician payment, access to care

## Abstract

**Background:**

Understanding all factors that affect a patient’s acute migraine treatment care is crucial. We sought to determine the impact of headache specialist density and the introduction of the gepants and lasmiditan on the prescription of acute treatments for migraine.

**Methods:**

We analyzed three scenarios: first, we performed linear regression analysis with the percentage of patients with migraine prescribed an acute medication in 2023, obtained via Epic Cosmos, and the density of headache specialists at the state level. Second, we conducted interrupted time-series analysis examining the change in patients prescribed the triptans before (2016–2019) and after (2020–2023) the introduction of the gepants and lasmiditan. Finally, we used regression analysis to look at the association of one pharmaceutical company, Pfizer, payments to physicians with prescriptions for that company’s gepant, rimegepant.

**Results:**

We included 6,559,854 patients with migraine and found that increased headache specialist density was associated with increased eletriptan, almotriptan, and naratriptan; there was no association with the other queried acute medications. In our interrupted time-series analysis, the introduction of the gepants and lasmiditan was linked to decreases in triptan utilization, except for eletriptan which remained stable, and rizatriptan which rose at a slower rate. Finally, increased Pfizer payments to physicians were associated with a higher percentage of patients prescribed rimegepant.

**Conclusion:**

Our study suggests increased headache provider availability is associated with more prescriptions for naratriptan, eletriptan, and almotriptan. Additionally, the introduction of the gepants and lasmiditan broadly decreased the utilization of triptans. Critically, there was a strong association between a pharmaceutical company’s, Pfizer, payments to physicians and utilization of their medication, rimegepant.

## 1 Introduction

Migraine-specific acute treatments are an integral component of a comprehensive treatment plan for patients with migraine (1). The introduction of the triptans in the 1990s revolutionized acute treatment for migraine. In 2019, lasmiditan was the first acute migraine medication approved by the Food and Drug Administration (FDA) since the approval of frovatriptan in 2001. The FDA approval of ubrogepant and rimegepant followed in December 2019 and February 2020, respectively. These newer treatments provided options for patients whose acute migraine medication needs were not met with triptans and non-steroidal anti-inflammatory drugs (NSAIDs). The gepants and lasmiditan have been presented as newer medications with improved tolerability.

In the setting of growing advances in migraine treatment, the field of headache medicine has experienced a steady growth of headache medicine fellowship training programs and an increase in the number of certified specialists, across the US, in the past two decades. Headache medicine specialists are certified by the United Council of Neurological Specialties (UCNS), with an initial cohort of specialists receiving certification in 2006. The four pathways for certification examination eligibility include: completing a headache medicine fellowship, practicing for a minimum of 36 months of which 25% of the time is spent with headache medicine cases, receiving a faculty appointment at a UCNS-accredited training program, and internationally training at a UCNS-accredited training program (2).

Our study examines the underexplored intersection between the introduction of lasmitidan and gepants and their impact on triptan prescription patterns, as well as, how the density of board-certified headache medicine specialists affected the prescription of acute migraine medications at the state level.

## 2 Methods

### 2.1 Study design

We conducted a retrospective study integrating multiple data sets: UCNS Headache Medicine certification records, Epic Cosmos patient data, Bureau of Labor Statistics, US Census data, and Open Payments data. This project was exempted from Institutional Review Board Review as it did not meet the criteria for Stanford University’s definition of Human Subject research requiring IRB approval per OHRP 45 CFR 46.102.

We first sought to determine how the presence of headache medicine specialists affected prescription patterns by correlating the density of UCNS headache medicine-certified physicians at the state level with the percentage of patients with migraine who received different acute medications.

The density of headache medicine specialists was calculated as the number of UCNS board-certified headache medicine physicians per 100,000 residents by state. The number of UCNS board-certified headache medicine physicians was obtained from the UCNS’ online listing of certified physicians as of August 2024 (3). All certified physicians were included regardless of date of certification and continuing certification status. Population data for 2023 was obtained from US Census data (4). Washington D.C. was not included as no headache medicine providers are registered as being in the district; those that practice in the area are logged as being in nearby states.

Prescription patterns were obtained from the Epic Cosmos research platform (Epic System Corporation, Verona, WI), an aggregated de-identified database of health systems’ electronic health records throughout the United States to be used for research (5). Data used in this study came from Epic Cosmos, a dataset created in collaboration with a community of Epic health systems representing more than 270 million patient records from over 1,568 hospitals and 35,000 clinics from all 50 states and Lebanon. Of note, the Epic Cosmos data is known to reflect the US populations demographics (6).

For this scenario, we created a population in Epic Cosmos that included all patients in the US who had received an International Classification of Diseases, Tenth Edition (ICD-10) code of G43 “Migraine,” or higher resolution in 2023. These patients were then divided by state of residence, to give the number of patients with a diagnosis of G43 or higher resolution by state. The G43 population was then queried for the number of patients who had been prescribed one of the seven triptans (sumatriptan, rizatriptan, eletriptan, naratriptan, almotriptan, frovatriptan, and zolmitriptan), two acute gepants (rimegepant and ubrogepant), or lasmiditan by state for the year 2023. Zavegepant was not included as it was released in mid-2023. These were then converted into percentages for each medication-state cell. Non-steroidal anti-inflammatory drugs and acetaminophen could not be included as Epic Cosmos does not reliably track over-the-counter medications.

Additionally, to ensure that the effect on prescription patterns was indeed due to the presence of headache specialists and not a marker of access to healthcare, we also sought to control for availability of physicians in general as well as insurance status. To control for the availability of physicians, we determined the density of family medicine physicians per 100,000 residents for each state using Bureau of Labor Statistics (BLS) data (7). We elected not to use neurologists per 100,000, as many states did not report this data to the BLS, and since the vast majority of headache specialists are neurologists, this would make the control variable not independent of the investigated variable. To control for insurance status, we used United States Census Bureau data for Health Insurance Coverage in the United States for 2022, the most recent available year, to obtain the percentage of patients with insurance by state (8).

To examine the impact of the gepants and lasmiditan introduction on the prescription of triptans, we sought to look at the percentage of patients with migraine who had received one of the above acute migraine medications by year for 2016–2023. These years were chosen so that they could be divided into two four-year segments divided by the clustered introduction of rimegepant, ubrogepant, and lasmiditan between October 2019 and February 2020: 2016–2019 as the pre-introduction years, and 2020–2023 as the post-introduction years. We then built a population defined by patients who had received an ICD-10 code of G43 and who had received a prescription for one of the acute migraine medications listed above as well as rimegepant, ubrogepant, and lasmiditan during 2016–2023. We then queried the database for the number of patients from this population prescribed each of the acute migraine medications by year and converted these to percentages. Subset analysis was also conducted for patients with a diagnosis of G43.7 “chronic migraine without aura” or higher resolution and for patients with a diagnosis of G44.4 “drug-induced headache” as a surrogate for medication-overuse headache (MOH) given that MOH does not have a dedicated ICD-10 code.

After conducting the original analysis and finding that headache provider density was not associated with the prescription of the novel gepants and lasmiditan, contrary to expectation, we sought to determine if the marketing campaigns for these medications could be associated with access to these medications. For this, we used the Center for Medicare and Medicaid Service’s Open Payments database to examine if there was an association between payments to physicians by state and the company’s novel medication. Specifically, we examined the association between Pfizer’s general payments to physicians and the percentage of patients with migraine prescribed rimegepant, which they produce, by state. Abbvie was not considered as they have multiple migraine medications on the market that have been available for a variable duration of time. Eli Lilly was not considered as lasmiditan did not have sufficient uptake as to gauge an effect. The Open Payments database was queried for 2023 Pfizer general payments, not research payments, to all recipients (including physicians, non-physicians, and teaching hospitals) and for all natures of payment including compensation for services other than consulting, including serving as faculty or as a speaker at a venue other than a continuing education program; food and beverage; consulting fees; grants; acquisitions; travel and lodging; space rental or facility fees; honoraria; education; royalty or license; compensation for serving as faculty or as a speaker for medical education program; charitable contributions; and entertainment (9). These were then aggregated to the state level. The percentage of patients with migraine who received rimegepant during 2023 was used from scenario one.

### 2.2 Statistical analysis

In the first scenario, we performed a linear regression using ordinary least squares with Stata 14, with the percentage of patients with migraine who received each of the acute migraine medications as the dependent variables run in separate regressions, and the density of headache providers as the independent variable. The density of family medicine providers and percentage of patients with insurance were controlled for in the regression.

In the second scenario, we used interrupted time series analysis with STATA, run for each of the seven triptans. The slope of the regression, the change in the percentage of patients with each triptan prescription per year, before and after the introduction of rimegepant, ubrogepant, and lasmiditan, were the outcome variables. The percentage of patients receiving each triptan by year was the independent variable with the years 2016–2019 denoted as the pre-intervention and 2020–2023 denoted as the post-intervention periods. This was conducted for all patients as well as for our subsets of patients with chronic migraine and MOH.

In the third scenario, we performed a linear regression using ordinary least squares with STATA 14, with the percentage of patients with migraine by state who received rimegepant as the dependent variable and with the amount of Pfizer general payments in dollars by state as the dependent variable. Outlier states with payments over $1,500,000 were dropped from analysis.

The map of the density of headache providers by state and the scatterplot with trendline for general payments by Pfizer and percentage of patients with migraine who received rimegepant were generated with Microsoft Excel.

This is the primary reporting of these data. Missing data was limited to the number of family medicine physicians for the state of Rhode Island and this state was dropped for the first scenario as a result. In instances where Epic Cosmos returned that “10 or fewer” patients were in a category, this was replaced by a zero. Statistical significance was set at *p* < 0.05.

## 3 Results

**Figure 1:**
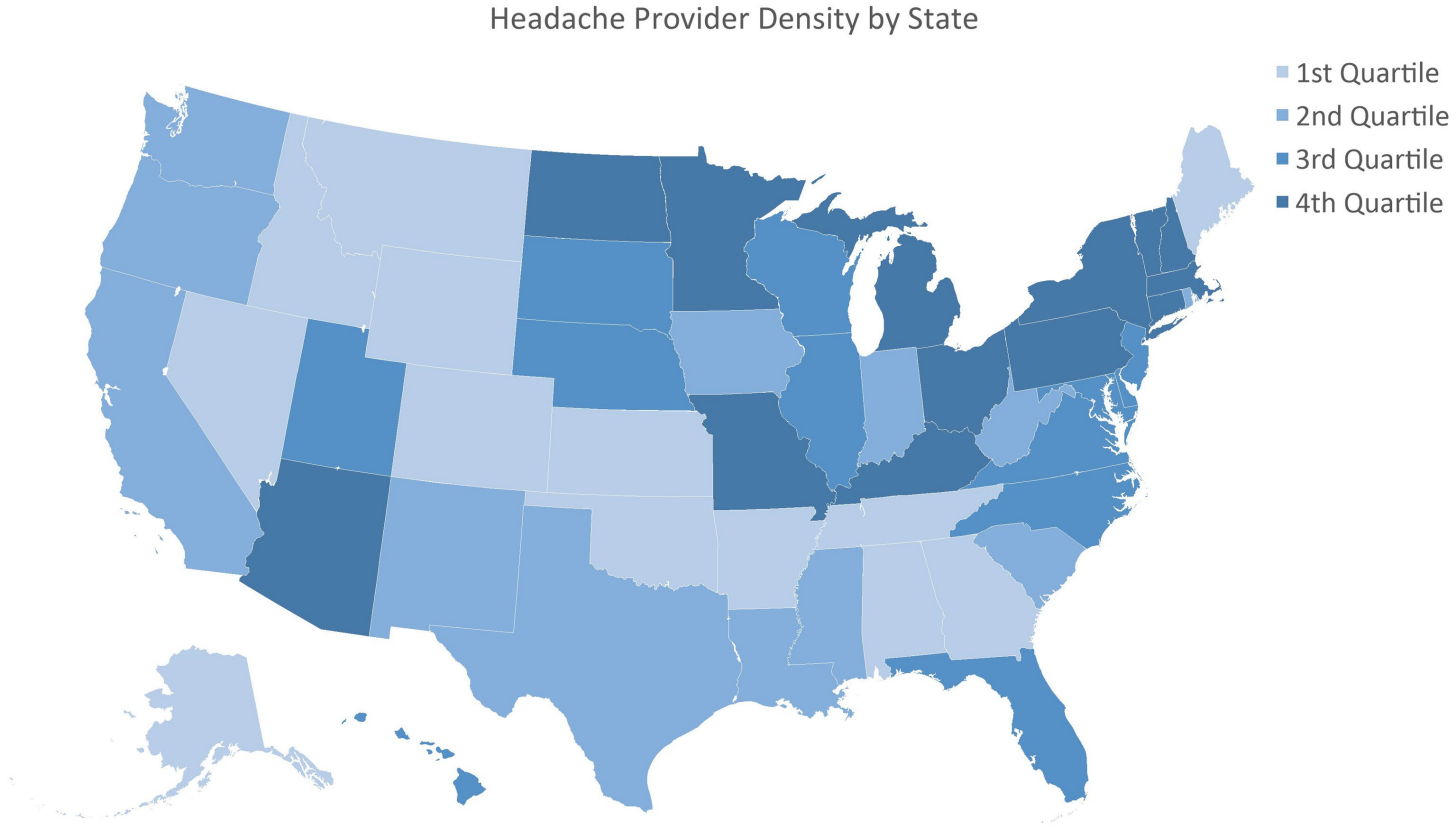
Headache provider density by state.

For the first scenario, 6,559,854 patients had a G43 diagnosis in 2023 and were included in the analysis, including all 50 states (Table 1). The states had an average of 0.21 ± 0.16 UCSN-certified headache medicine physicians per 100,000 (Figure 1), 24.04 ± 16.77 family medicine physicians per 100,000 and insurance rates of 93.59 ± 2.69%.

**Table 1:** Demographics and medications prescribed for all included patients for 2016–2023.

		2016 (*N* = 2,830,911)	2017 (*N* = 3,364,597)	2018 (*N* = 3,903,389)	2019 (*N* = 4,404,779)	2020 (*N* = 4,714,942)	2021 (*N* = 5,323,038)	2022 (*N* = 5,899,095)	2023 (*N* = 6,559,854)
Average AGE (in years) ± St. Dev		50 ± 17	50 ± 17	49 ± 17	49 ± 17	48 ± 17	48 ± 17	47 ± 17	47 ± 17
Percent female		2,277,068 (80.4%)	2,701,634 (80.3%)	3,128,507 (80.1%)	3,530,649 (80.2%)	3,797,752 (80.5%)	4,283,993 (80.5%)	4,743,177 (80.4%)	5,267,784 (80.3%)
Race									
	American Indian or Alaska Native	31,585 (1.1%)	38,677 (1.1%)	45,507 (1.2%)	52,383 (1.2%)	57,132 (1.2%)	65,445 (1.2%)	73,000 (1.2%)	81,428 (1.2%)
	Asian	59,638 (2.1%)	72,960 (2.2%)	86,914 (2.2%)	100,610 (2.3%)	110,253 (2.3%)	129,447 (2.4%)	146,143 (2.5%)	165,539 (2.5%)
	Black or African American	332,059 (11.7%)	396,330 (11.8%)	463,412 (11.9%)	525,928 (11.9%)	553,062 (11.7%)	633,528 (11.9%)	707,707 (12%)	797,601 (12.2%)
	Native Hawaiian or Other Pacific Islander	12,215 (0.4%)	14,467 (0.4%)	16,720 (0.4%)	19,082 (0.4%)	20,608 (0.4%)	23,398 (0.4%)	26,186 (0.4%)	29,237 (0.4%)
	Other	322,688 (11.4%)	391,655 (11.6%)	461,684 (11.8%)	530,519 (12%)	577,030 (12.2%)	669,644 (12.6%)	763,394 (12.9%)	868,917 (13.2%)
	White	2,314,627 (81.8%)	2,745,625 (81.6%)	3,179,777 (81.5%)	3,580,829 (81.3%)	3,838,492 (81.4%)	4,316,493 (81.1%)	4,767,000 (80.8%)	5,275,871 (80.4%)
Ethnicity									
	Hispanic or Latino	205,175 (7.2%)	252,257 (7.5%)	303,119 (7.8%)	351,667 (8%)	384,078 (8.1%)	447,366 (8.4%)	513,727 (8.7%)	590,483 (9%)
	Not Hispanic or Latino	2,452,993 (86.7%)	2,909,609 (86.5%)	3,368,043 (86.3%)	3,791,381 (86.1%)	4,049,377 (85.9%)	4,555,198 (85.6%)	5,026,609 (85.2%)	5,563,591 (84.8%)
	None of the above	172,743 (6.1%)	202,731 (6%)	232,227 (5.9%)	261,731 (5.9%)	281,487 (6%)	320,474 (6%)	358,759 (6.1%)	405,780 (6.2%)
Any acute medication		629,467	794,446	969,186	1,144,045	1,321,627	1,600,005	1,883,900	2,216,064
Sumatriptan		432,628 (68.7%)	542,009 (68.2%)	657,140 (67.8%)	769,236 (67.2%)	854,000 (64.6%)	988,264 (61.7%)	1,109,355 (58.8%)	1,256,439 (56.6%)
Rizatriptan		149,380 (23.7%)	196,360 (24.7%)	249,941 (25.7%)	307,060 (26.8%)	360,766 (27.2%)	442,295 (27.6%)	523,381 (27.7%)	621,525 (28.0%)
Eletriptan		44,727 (7.1%)	52,653 (6.6%)	60,471 (6.2%)	68,123 (5.9%)	74,584 (5.6%)	83,550 (5.2%)	90,630 (4.8%)	98,584 (4.4%)
Almotriptan		5,610 (0.8%)	6,762 (0.8%)	7,852 (0.8%)	8,775 (0.7%)	9,106 (0.6%)	9,726 (0.6%)	10,302 (0.5%)	10,827 (0.4%)
Zolmitriptan		35,444 (5.6%)	44,201 (5.5%)	52,500 (5.4%)	59,307 (5.1%)	63,158 (4.7%)	68,492 (4.2%)	73,862 (3.9%)	79,828 (3.6%)
Frovatriptan		9,013 (1.4%)	10,999 (1.3%)	12,874 (1.3%)	14,357 (1.2%)	15,348 (1.1%)	16,352 (1.0%)	17,046 (0.9%)	17,906 (0.8%)
Naratriptan		22,415 (3.5%)	29,335 (3.6%)	35,367 (3.6%)	41,703 (3.6%)	47,998 (3.6%)	56,425 (3.5%)	63,607 (3.3%)	71,776 (3.2%)
Rimegepant		0 (0%)	0 (0%)	0 (0%)	0 (0%)	34,146 (2.5%)	114,987 (7.1%)	205,077 (10.8%)	302,126 (13.%)
Ubrogepant		0 (0%)	0 (0%)	0 (0%)	0 (0%)	51,502 (3.8%)	108,059 (6.7%)	161,774 (8.5%)	226,611 (10.%)
Lasmiditan		0 (0%)	0 (0%)	0 (0%)	0 (0%)	3,315 (0.2%)	6,617 (0.4%)	8,720 (0.4%)	10,469 (0.4%)

After controlling for availability of providers overall and insurance status, a higher density of headache medicine providers was associated with increased utilization of eletriptan (Coefficient 3.58; 95% CI: 1.92–6.14, *p* = 0.007), almotriptan (Coefficient 0.58, 95% CI: 0.20 to 0.97, *p* = 0.004) and naratriptan (coefficient 4.24; 95% CI: 1.42 to 7.07, *p* = 0.004) (Table 2). Prescriptions for any acute medications, as well as the other triptans, lasmiditan, rimegepant, and ubrogepant, did not show a statistically significant association with headache provider density.

**Table 2:** Regression coefficients for impact of headache specialists on triptan presciptions.

	Initial regression			Controlling for family medicine providers			Controlling for insurance coverage			Controlling for both factors		
Medication	Coefficient	95% CI	*p*-value	Coefficient	95% CI	*p*-value	Coefficient	95% CI	*p*-value	Coefficient	95% CI	*p*-value
Any of these	−6.99	−15.13 to 1.15	0.091	−7.79	−16.31 to 0.73	0.072	−3.88	−13.0 to 5.24	0.396	−4.67	−14.20 to 4.85	0.328
Sumatriptan	2.90	−9.08 to 14.89	0.628	2.66	−9.94 to 15.2	0.673	−5.13	−17.89 to 7.63	0.423	−5.61	−18.98 to 7.76	0.403
Rizatriptan	−5.50	12.57 to 1.56	0.124	−6.11	13.5 to 12.9	0.103	−3.36	−11.35 to 4.62	0.401	−3.97	−12.3 to 4.38	0.344
Eletriptan	2.39	0.18 to 4.59	**0.034**	2.59	0.28 to 4.90	**0.029**	3.34	0.89 to 5.80	**0.009**	3.58	1.02 to 6.14	**0.007**
Almotriptan	0.65	0.28 to 1.01	**0.001**	0.78	0.43 to 1.14	**<0.001**	0.44	0.05 to 0.84	**0.029**	0.58	0.20 to 0.97	**0.004**
Naratriptan	4.16	1.76 to 6.56	**0.001**	4.60	2.12 to 7.08	**0.001**	3.78	1.04 to 6.52	**0.008**	4.24	1.42 to 7.07	**0.004**
Frovatriptan	0.37	−0.20 to 0.93	0.198	0.44	−0.15 to 1.03	0.141	0.25	−0.39 to 0.89	0.437	0.32	−0.34 to 0.99	0.333
Zolmitriptan	0.84	−1.16 to 2.85	0.402	0.52	−1.56 to 2.60	0.616	0.50	−1.79 to 2.79	0.663	0.14	−2.23 to 2.51	0.905
Rimegepant	−0.12	−8.06 to 7.81	0.975	0.18	−8.16 to 8.52	0.966	3.48	−5.32 to 12.3	0.43	3.91	−0.53 to 13.13	0.398
Ubrogepant	0.27	−7.33 to 7.87	0.944	0.60	−7.38 to 8.59	0.88	4.43	−3.88 to 12.73	0.289	4.90	−3.80 to 13.60	0.262
Lasmiditan	−0.15	−0.73 to 0.44	0.612	−0.29	−0.88 to 0.31	0.334	0.06	−0.60 to 0.71	0.861	−0.09	−0.76 to 0.58	0.788

Bold indicates *p* < 0.05.

For the second scenario, Table 1 shows the number of patients per year with a diagnosis of G43 Migraine who received acute migraine medication prescription during the study time frame. Age decreased over time from 50 ± 17 in 2016 to 47 ± 17 to 2023 but female sex was unchanged. Prior to the introduction of the two gepants and lasmiditan, sumatriptan, eletriptan, almotriptan, frovatriptan and zolmitriptan all showed statistically significant decreases in their use over time (Table 3). Naratriptan had stable prescriptions during this time period. Notably, rizatriptan was the only triptan to have an increase in utilization during this period. The introduction of the new medications largely accelerated the decline in triptan prescriptions; sumatriptan, naratriptan, almotriptan, naratriptan, frovatriptan, and zolmitriptan showed faster rates of decrease while only eletriptan’s rate of decrease was unaffected. Rizatriptan continued to increase its rate of utilization but at a slower rate (Pre-introduction rate: +1.03%/year; 95% CI 0.96–1.10%, *p* < 0.001; Post-introduction effect: −0.79%/year; −0.89 to −0.69, *p* < 0.001).

**Table 3:** Coefficients for interrupted time series analysis for pre-gepant/ditan and post-gepant/ditan periods.

	Pre-gepant/ditan			Post-gepant/ditan		
Triptan	Coefficient	95% CI	*p*-value	Coefficient	95% CI	*p*-value
All migraine						
Sumatriptan	−0.47	−0.70 to −0.22	**0.006**	−2.17	−2.51 to −1.82	**<0.001**
Rizatriptan	1.03	0.96 to 1.10	**<0.001**	−0.79	−0.89 to −0.69	**<0.001**
Eletriptan	−0.41	−0.49 to −0.32	**<0.001**	0.01	−0.11 to 0.14	0.809
Naratriptan	0.02	−0.01 to 0.04	0.200	−0.14	−0.18 to −0.10	**0.001**
Almotriptan	−0.04	−0.05 to ‘−0.03	**<0.001**	-0.03	−0.04 to −0.02	**0.002**
Frovatriptan	−0.07	−0.08 to 0.05	**<0.001**	−0.05	−0.07 to −0.03	**0.002**
Zolmitriptan	−0.18	−0.25 to −0.11	**0.002**	−0.22	−0.32 to −0.11	**0.004**
Chronic migraine						
Sumatriptan	−0.77	−1.42 to −0.11	**0.031**	−2.59	−0.3.51 to −1.66	**0.001**
Rizatriptan	1.28	1.15 to 1.40	**<0.001**	−1.73	−1.91 to −1.55	**<0.001**
Eletriptan	−0.45	−0.60 to −0.29	**0.001**	−0.13	−0.35 to 0.08	0.160
Naratriptan	−0.14	−0.25 to −0.02	**0.029**	−0.30	−0.46 to −0.14	**0.007**
Almotriptan	−0.002	−0.03 to 0.02	0.846	−0.13	−0.17 to −0.08	**0.002**
Frovatriptan	−0.16	−0.24 to ‘-0.10	**0.003**	−0.05	−0.15 to 0.05	**0.244**
Zolmitriptan	−0.27	−0.49 to 0.04	**0.029**	−0.47	−0.79 to 0.15	**0.015**
Medication-overuse headache						
Sumatriptan	−1.41	−1.99 to −0.82	**0.003**	−1.66	−2.48 to −0.83	**0.005**
Rizatriptan	1.86	1.56 to 2.17	**<0.001**	−2.23	−2.68 to −1.83	**<0.001**
Eletriptan	−0.47	−0.78 to 0.17	**0.012**	−0.06	−0.49 to 0.37	0.705
Naratriptan	0.07	−0.13 to 0.28	0.379	−0.60	−0.89 to −0.31	**0.005**
Almotriptan	0.07	0.01 to 0.14	**0.033**	−0.22	−0.31 to −0.13	**0.003**
Frovatriptan	−0.09	−0.18 to ‘-0.01	**0.037**	−0.14	−0.26 to −0.02	**0.032**
Zolmitriptan	−0.26	−0.69 to 0.16	0.158	−0.52	−1.11 to 0.08	0.075

Bold indicates *p* < 0.05.

We then conducted subset analysis for the change in triptan prescriptions after the introduction of the gepants and lasmiditan for patients with chronic migraine and MOH and similar patterns were observed except for rizatriptan where in both subsets the post-introduction decreases in rizatriptan utilization outweighed the pre-introduction increase trend yielding a net decrease (Supplementary Tables 1, 2).

**Figure 2:**
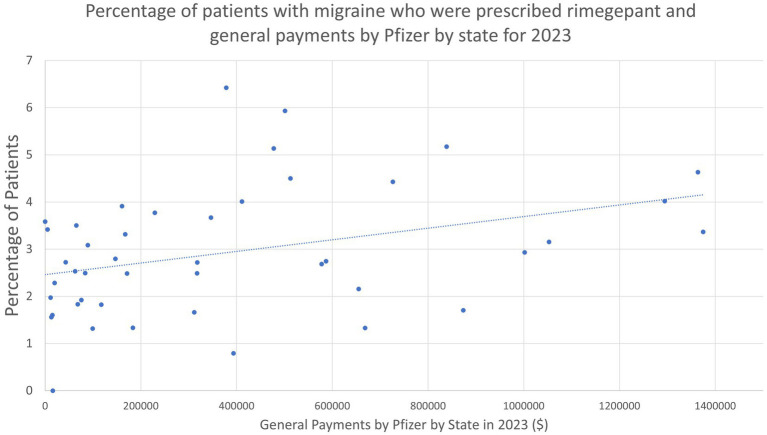
Percentage of patients with migraine who were prescribed rimegepant and general payments by Pfizer by state for 2023.

Given the unexpected finding that the density of headache providers was unrelated to the percentage of patients who received rimegepant, ubrogepant, or lasmiditan, we sought to investigate this further and queried whether pharmaceutical company spending could account for this. We observed a statistically significant association in Pfizer general payments by state and the state’s percent of patients with migraine who received rimegepant (Coefficient as percentage point increase in patients who used rimegepant per $100,000 spent: 0.123; 95% CI: 0.022–0.224, *p* < 0.001) (Figure 2).

## 4 Discussion

Providing optimal care for patients with migraine necessitates matching them with medications that are going to be effective and well-tolerated. Understanding the impact of access to specialty care and the availability of new medications is crucial and under-explored.

There are few studies examining the effect of specialist care on the treatment of disease, and our study adds a critical understanding for how patients access medications for the acute treatment of migraine. In most models of care, patients initially present to primary care physicians, and in the case of migraine, this likely translates to recommendations for either NSAIDs or the most common triptans, sumatriptan and rizatriptan. In our study, sumatriptan and rizatriptan were not linked to headache specialist density. Beyond these initial steps, patients can take a variety of different routes through the healthcare system to receive more advanced healthcare.

When patients are not able to establish with either a general neurologist or a headache specialist, we would expect that that they may not have access to treatments such as lasmiditan, gepants, or certain triptans. Reassuringly, our data do not support this occurring. Rather, only eletriptan, naratriptan, and almotriptan were linked to headache provider density, suggesting that sumatriptan, rizatriptan, zolmitriptan, frovatriptan, lasmiditan, rimegepant, and ubrogepant were accessible to patients independent of headache provider density. Our results suggest that additional headache provider capacity facilitated the prescription of eletriptan, naratriptan, and almotriptan, which may fill specific roles in the patient’s headache care.

While access to sumatriptan and rizatriptan being independent of headache medicine provider density was not unexpected, we did not expect access to gepants and lasmitidan to be independent of headache provider density. We also did not expect prescriptions for zolmitriptan and frovatriptan to be independent of headache medicine provider density given their special characteristics (nasal spray formulation for zolmitriptan and long half-life of frovatriptan).

The broad decreases in triptan prescriptions after the introduction of the gepants and lasmiditan were of generally similar magnitudes with the notable exception of sumatriptan which experienced a much larger decrease. Our study was not designed to assess the underpinning reasons for this accelerated decline but given sumatriptan’s ubiquity coupled with it often having the most side effects of the triptans, there was certainly the potential for a larger shift away from sumatriptan (10, 11). Similarly the other declines from the triptans are likely due to their worse side effect profiles relative to the gepants (12).

For rimegepant, our data suggest a notable association with prescription volume and pharmaceutical marketing campaigns. On the provider level, we observed a strong correlation between Pfizer payments to physicians and hospitals in a state and increased prescribing of rimegepant. Previous work suggests that pharmaceutical payments to physicians are associated with increased prescribing of medications broadly and that the prescribing of medication is more driven by the physician than the patient (13, 14). In 2023, Pfizer spent $177.9 million on advertising directly to patients for rimegepant, including using top celebrities in their campaign, such that patients may ask their PCP or general neurologist for the prescription (15). Additionally, the companies that produce rimegepant and ubrogepant have engaged in programs to provide samples to offices, which is also known to increase utilization of medications, including at the expense of using first line preferred medications (16, 17). These companies have also used copay assistance programs which there is some evidence to suggest also boosts usage of these medications (18). However, the Epic Cosmos data is prescription data only and does not mean that the medications were filled and started. As a result, it is also possible that receiving a prescription for the medications is not limited by access to headache medicine specialists, but this does not answer whether patients actually started these medications.

For lasmiditan, the lack of effect of headache provider density on its utilization likely stems from its overall low utilization; in 2023, only 0.3% of patients in our study with an acute medication prescription received one for lasmiditan and this was stable from 2022. Reasons for this could potentially include lasmiditan’s comparatively poor side effect profile (12). Additionally, as a controlled substance, access to lasmiditan can also be felt to be more difficult in comparison to other medications.

There are few studies on the impact of a new generation of medications on older ones, but our study shows that the newer medications broadly decreased utilization of the triptans. This somewhat differs from a previous study of new medications for depression in New Zealand where they were prescribed additionally and not as substitutes (19). Unlike the adjunct use of new anti-depressants, the gepants and lasmiditan provided options for patients whose acute migraine medication needs were not met with triptans and non-steroidal anti-inflammatory drugs (NSAIDs); one study of 10,509 patients in 2020 showed that fewer than half of the patients in the study refilled their first triptan prescription within 12 months, and 90.5% of the patients only tried one triptan, suggesting that the triptans were not fulfilling the role of an acute migraine medication for many patients (20). This is likely due to their comparatively poor side effect profile, with a 2017 network meta-analysis noting sumatriptan, rizatriptan, and zolmitriptan having higher rates of all-adverse events relative to placebo (11). In contrast, the gepants and lasmiditan have been presented as newer medications with improved tolerability with a 2021 meta-analysis substantiating this claim for ubrogepant and rimegepant but demonstrating an increased risk of adverse events for lasmiditan relative to placebo (1). Additionally, the gepants do not contribute to medication-overuse headache and can be an option for patients with both migraine and medication-overuse headache (21).

However, the gepants and lasmiditan are significantly more expensive without higher efficacy, and their substitution represents an overall shift toward higher costs for the health care system. For example, sumatriptan can be $25.14 per 100 mg tablet while rimegepant can be $149.85 per tablet, such that a shift from sumatriptan to rimegepant is a ~ 6-fold increase in cost (22, 23). Additionally, the role of pharmaceutical companies in this cannot be overlooked as our work suggests that payments to physicians propped up prescriptions above what they would naturally be. This shift is also crucial given that the gepants and lasmiditan are also CYP3A4 substrates and can interact with many other medications in a way that the triptans do not. This is also important given that patients with migraine often have other comorbidities that require other medications as well (24).

However, the pharmaceutical payments data alone does not provide a sufficient explanation for why there is a lack of association between headache providers and the majority of the acute medications for migraine. Unfortunately, the Epic Cosmos data utilized could not give provider or specialty information such that this could not be assessed with our current data set. Future studies could use information such as physician surveys to understand the motivation for physicians, including those of differing specialties, to prescribe specific medications for the acute treatment of migraine over others. Additionally, while our studies assess direct payment data, samples to physicians are not similarly tracked and so it is difficult to objectively measure this. Consequently, a survey of physician preferences would also need to account for what, if any, samples of medications they received.

Our study has multiple limitations. Critically, the extracted prescriptions are ones that have been written and not those that have been filled such that it is quite possible the outcomes are different when assessing what medications patients were actually able to start; given the many insurance plans require step therapy and prior authorizations for the gepants and lasmiditan, it is unlikely that all patients who received a prescription for these medications successfully picked them up from their pharmacy. Additionally, the Epic Cosmos data is, by definition, only extracted from health systems who use Epic. Given that the Epic EHR can be quite costly, there may be a selection bias at play for the patients who are seen in facilities that use Epic. Epic Cosmos was also rolled out over the study period for the assessment of the impact of the gepants on the triptans. While we attempted to control for this by looking at percentages to adjust for the variable number of patients seen yearly, it is possible that the access-to-care profile is different for early adopters and late adopters. Moreover, we defined our groups by ICD-10 codes for migraine and were not able to define them by strict ICHD-3 criteria. Similarly, our MOH group had to be defined by the ICD-10 code G44.4 “drug-induced headache” which may be an suboptimal surrogate for MOH. Episodic migraine too is not included in the ICD-10 and so could not be positively defined and assessed. We also elected not to assess migraine with aura as a subset as though it does have a discrete ICD-10 code, we were concerned about the validity of a diagnosis of migraine aura in a cohort drawn from all providers such that the results would be difficult to interpret.

Moreover, we did see a statistically significant change in the average age of a patient who received an acute medication during the study. That said, it is unclear what clinical impact a shift from an average of 50 years old to 46 years old has on our study. Beyond this, given that the gepants and lasmiditan do not have the contraindications that the triptans do, it may have been expected that average age would have increased over time as older patients with more comorbidities were able to get access to acute migraine medications.

Our work suggests that headache provider availability may not be a barrier to access to acute migraine medications. Headache provider availability can expand access to naratriptan, eletriptan, and almotriptan, but not to the other triptans, gepants, or lasmiditan. Given that these three triptans can be quite beneficial for certain groups, next steps would include educational campaigns with PCPs and general neurologists for helping connect patients with these triptans. Similarly, robust e-Consult programs that provide specific recommendations can help bridge this divide as well. Additionally, while the gepants and lasmiditan decreased usage of the triptans in our study time frame, future studies can reassess this impact as physician payments, advertising, samples, and copays decrease over time.

## Data Availability

The datasets presented in this article are not readily available because Epic Cosmos data is available to individuals whose institutions have permitted access to Epic Cosmos and completed appropriate training. The data usage agreement for Epic Cosmos does not permit unauthorized users from accessing the data. All other used datasets are publicly available at the linked sources. Requests to access the datasets should be directed to https://cosmos.epic.com/.

## References

[1] Puledda F Sacco S Diener HC Ashina M Al-Khazali HM Ashina S. International headache society global practice recommendations for the acute pharmacological treatment of migraine. Cephalalgia. (2024) 44:03331024241252666. doi: 10.1177/03331024241252666, PMID: 39133176

[2] Headache Medicine Certification Eligibility Criteria. Available at: https://www.ucns.org/common/Uploaded%20files/Certification/Headache%20Medicine%20Examination%20Eligibility%20Criteria.pdf (Accessed September 4, 2024).

[3] Headache Medicine Diplomate Directory. Available at: https://www.ucns.org/Online/Online/Diplomate_Directory.aspx?hkey=f8f00552-f924-4ef6-a9bb-6023b1cd341b (Accessed September 4, 2024).

[4] Bureau UC. State population totals and components of change: 2020–2023. (2024). Available at: https://www.census.gov/data/tables/time-series/demo/popest/2020s-state-total.html (Accessed September 4, 2024).

[5] Tarabichi Y Frees A Honeywell S Huang C Naidech AM Moore JH. The Cosmos collaborative: a vendor-facilitated electronic health record data aggregation platform. ACI open. (2021) 5:e36–46. doi: 10.1055/s-0041-1731004, PMID: 35071993 PMC8775787

[6] About. Epic Cosmos. Available at: https://cosmos.epic.com/about/ (Accessed September 4, 2024).

[7] Bureau of Labor Statistics. U.S. Bureau of Labor Statistics. Available at: https://data.bls.gov/oes/#/home

[8] Keisler-Starkey K. Health insurance coverage in the United States: 2022 (2023). Available at: https://www.census.gov/library/publications/2023/demo/p60-281.html (Accessed September 4, 2024).

[9] CMS Open Payments for Pfizer. Available at: https://openpaymentsdata.cms.gov/company/100000000286 (Accessed September 4, 2024).

[10] Fox AW. Comparative tolerability of oral 5-HT1B/1D agonists. Headache. (2000) 40:521–7. doi: 10.1111/j.1526-4610.2000.00083.x 10940090

[11] Thorlund K Toor K Wu P Chan K Druyts E Ramos E. Comparative tolerability of treatments for acute migraine: a network meta-analysis. Cephalalgia. (2017) 37:965–78. doi: 10.1177/0333102416660552, PMID: 27521843

[12] Yang CP Liang CS Chang CM Yang CC Shih PH Yau YC. Comparison of new pharmacologic agents with triptans for treatment of migraine: a systematic review and meta-analysis. JAMA Netw Open. (2021) 4:e2128544-.CMS. doi: 10.1001/jamanetworkopen.2021.28544, PMID: 34633423 PMC8506232

[13] Mitchell AP Trivedi NU Gennarelli RL Chimonas S Tabatabai SM Goldberg J. Are financial payments from the pharmaceutical industry associated with physician prescribing? A systematic review. Ann Intern Med. (2021) 174:353–61. doi: 10.7326/M20-5665, PMID: 33226858 PMC8315858

[14] Cutler D Skinner JS Stern AD Wennberg D. Physician beliefs and patient preferences: a new look at regional variation in health care spending. Am Econ J Econ Pol. (2019) 11:192–221. doi: 10.1257/pol.20150421, PMID: 32843911 PMC7444804

[15] Gil B. Ozempic and 9 more of big Pharma’s most-advertised drugs. Quartz; (2024). Available at: https://qz.com/ozempic-skyrizi-pharma-drug-advertising-spending-1851565047 (Accessed September 4, 2024).

[16] Symm B Averitt M Forjuoh SN Preece C. Effects of using free sample medications on the prescribing practices of family physicians. J Am Board Fam Med. (2006) 19:443–9. doi: 10.3122/jabfm.19.5.443, PMID: 16951293

[17] Pinckney RG Helminski AS Kennedy AG Maclean CD Hurowitz L Cote E. The effect of medication samples on self-reported prescribing practices: a statewide, cross-sectional survey. J Gen Intern Med. (2011) 26:40–4. doi: 10.1007/s11606-010-1483-x, PMID: 20809157 PMC3024102

[18] Parekh KD Wong WB Zullig LL. Impact of co-pay assistance on patient, clinical, and economic outcomes. Am J Manag Care. (2022) 28:e189–97. doi: 10.37765/ajmc.2022.89151, PMID: 35546593

[19] Roberts E Norris P. Growth and change in the prescribing of anti-depressants in New Zealand: 1993-1997. N Z Med J. (2001) 114:25–7. PMID: 11277470

[20] Lipton RB Marcus SC Shewale AR Dodick DW Viswanathan HN Doshi JA. Acute treatment patterns in patients with migraine newly initiating a triptan. Cephalalgia. (2020) 40:437–47. doi: 10.1177/0333102420905307, PMID: 32138526 PMC7160749

[21] Lo Castro F Guerzoni S Pellesi L. Safety and risk of medication overuse headache in lasmiditan and second-generation gepants: a rapid review. Drug Healthcare Patient Safety. (2021) 13:233–40. doi: 10.2147/DHPS.S304373, PMID: 34849034 PMC8627250

[22] Sumatriptan: Drug Information. Available at: https://www.uptodate.com/contents/sumatriptan-drug-information?source=auto_suggest&selectedTitle=1~2---1~2---sumatriptan&search=sumatriptan (Accessed September 4, 2024).

[23] Rimegepant: Drug Information. Available at: https://www.uptodate.com/contents/rimegepant-drug-information?sectionName=Adult&topicId=127256&search=rimegepant&usage_type=panel&anchor=F54182781&source=panel_search_result&selectedTitle=1%7E5&showDrugLabel=true&kp_tab=drug_general&display_rank=1#F54182781 (Accessed September 4, 2024).

[24] Lipton RB Fanning KM Buse DC Martin VT Hohaia LB Adams AM. Migraine progression in subgroups of migraine based on comorbidities: results of the CaMEO study. Neurology. (2019) 93:e2224–36. doi: 10.1212/WNL.0000000000008589, PMID: 31690685 PMC6937494

