# Supplementary material for: The impact of headache specialist density and the introduction of gepants and lasmitidan on prescriptions for acute migraine treatments: a regression and interrupted time series analysis
Source: Front Neurol. 2025 Feb 7;16:1530499. doi: 10.3389/fneur.2025.1530499 (PMC11843553; doi:10.3389/fneur.2025.1530499)
Supplement: Supplementary file 1 [file Table_1.DOCX]

Supplementary Table 1. Demographics and medications prescribed for included patients with chronic migraine for 2016-2023

|  |  | 2016 (N=220667) | 2017 (N=303110) | 2018 (N=386416) | 2019 (N=458516) | 2020 (N=515664) | 2021 (N=607638) | 2022 (N=676496) | 2023 (N=776924) |
| --- | --- | --- | --- | --- | --- | --- | --- | --- | --- |
| Average Age (in years)±St. Dev |  | 51±16 | 50±16 | 50±16 | 49±16 | 49±16 | 48±16 | 48±16 | 47±16 |
| Percent Female |  | 184692 (83.7%) | 253798 (83.7%) | 323706 (83.8%) | 384043 (83.8%) | 433215 (84%) | 510720 (84.1%) | 567999 (84%) | 651651 (83.9%) |
| Race |  |  |  |  |  |  |  |  |  |
|  | American Indian or Alaska Native | 2796 (1.3%) | 3895 (1.3%) | 5013 (1.3%) | 6107 (1.3%) | 7163 (1.4%) | 8554 (1.4%) | 9573 (1.4%) | 10873 (1.4%) |
|  | Asian | 4043 (1.8%) | 5639 (1.9%) | 7486 (1.9%) | 9138 (2%) | 10564 (2%) | 13068 (2.2%) | 14902 (2.2%) | 17597 (2.3%) |
|  | Black or African American | 24792 (11.2%) | 33788 (11.1%) | 42845 (11.1%) | 51513 (11.2%) | 58131 (11.3%) | 69632 (11.5%) | 78192 (11.6%) | 91008 (11.7%) |
|  | Native Hawaiian or Other Pacific Islander | 833 (0.4%) | 1139 (0.4%) | 1468 (0.4%) | 1877 (0.4%) | 2058 (0.4%) | 2507 (0.4%) | 2703 (0.4%) | 3193 (0.4%) |
|  | Other | 23630 (10.7%) | 33463 (11%) | 43412 (11.2%) | 52367 (11.4%) | 60268 (11.7%) | 73777 (12.1%) | 85309 (12.6%) | 100476 (12.9%) |
|  | White | 185424 (84%) | 254560 (84%) | 324133 (83.9%) | 383852 (83.7%) | 431367 (83.7%) | 505849 (83.2%) | 561465 (83%) | 642073 (82.6%) |
| Ethnicity |  |  |  |  |  |  |  |  |  |
|  | Hispanic or Latino | 13964 (6.3%) | 20083 (6.6%) | 26969 (7%) | 32774 (7.1%) | 37895 (7.3%) | 46094 (7.6%) | 53266 (7.9%) | 63551 (8.2%) |
|  | Not Hispanic or Latino | 197146 (89.3%) | 269537 (88.9%) | 342156 (88.5%) | 405157 (88.4%) | 454168 (88.1%) | 533085 (87.7%) | 590840 (87.3%) | 674750 (86.8%) |
|  | None of the above | 9557 (4.3%) | 13490 (4.5%) | 17291 (4.5%) | 20585 (4.5%) | 23601 (4.6%) | 28459 (4.7%) | 32390 (4.8%) | 38623 (5%) |
| Medications |  |  |  |  |  |  |  |  |  |
| Any Acute Medication |  | 97061 | 140090 | 184111 | 223040 | 271534 | 342542 | 397312 | 474081 |
| Sumatriptan |  | 62243 (64.1%) | 88634 (63.3%) | 115457 (62.7%) | 137721 (61.7%) | 152909 (56.3%) | 178310 (52.1%) | 192715 (48.5%) | 219537 (46.3%) |
| Rizatriptan |  | 28198 (29.1%) | 42584 (30.4%) | 58387 (31.7%) | 73317 (32.9%) | 87225 (32.1%) | 108805 (31.8%) | 123504 (31.1%) | 146229 (30.8%) |
| Eletriptan |  | 9877 (10.2%) | 13335 (9.5%) | 16641 (9%) | 19737 (8.8%) | 22087 (8.1%) | 25618 (7.5%) | 27378 (6.9%) | 30308 (6.4%) |
| Almotriptan |  | 1373 (1.4%) | 1995 (1.4%) | 2657 (1.4%) | 3123 (1.4%) | 3132 (1.2%) | 3320 (1%) | 3478 (0.9%) | 3591 (0.8%) |
| Zolmitriptan |  | 8558 (8.8%) | 12299 (8.8%) | 15466 (8.4%) | 17950 (8%) | 19012 (7%) | 20223 (5.9%) | 20810 (5.2%) | 22570 (4.8%) |
| Frovatriptan |  | 2664 (2.7%) | 3581 (2.6%) | 4313 (2.3%) | 5029 (2.3%) | 5282 (1.9%) | 5505 (1.6%) | 5660 (1.4%) | 6057 (1.3%) |
| Naratriptan |  | 8350 (8.6%) | 11862 (8.5%) | 14994 (8.1%) | 18395 (8.2%) | 20956 (7.7%) | 25005 (7.3%) | 27045 (6.8%) | 30417 (6.4%) |
| Rimegepant |  | 0 (0%) | 0 (0%) | 0 (0%) | 0 (0%) | 21222 (7.8%) | 60682 (17.7%) | 91477 (23%) | 122900 (25.9%) |
| Ubrogepant |  | 0 (0%) | 0 (0%) | 0 (0%) | 0 (0%) | 29538 (10.9%) | 52667 (15.4%) | 69864 (17.6%) | 93776 (19.8%) |
| Lasmiditan |  | 0 (0%) | 0 (0%) | 0 (0%) | 0 (0%) | 2839 (1%) | 5131 (1.5%) | 6327 (1.6%) | 7573 (1.6%) |
