# Supplementary material for: The impact of headache specialist density and the introduction of gepants and lasmitidan on prescriptions for acute migraine treatments: a regression and interrupted time series analysis
Source: Front Neurol. 2025 Feb 7;16:1530499. doi: 10.3389/fneur.2025.1530499 (PMC11843553; doi:10.3389/fneur.2025.1530499)
Supplement: Supplementary file 2 [file Table_2.DOCX]

Supplementary Table 2. Demographics and medications prescribed for included patients with medication-overuse headache for 2016-2023

|  |  | 2016 (N=29429) | 2017 (N=37805) | 2018 (N=44618) | 2019 (N=51707) | 2020 (N=54717) | 2021 (N=61194) | 2022 (N=65148) | 2023 (N=69723) |
| --- | --- | --- | --- | --- | --- | --- | --- | --- | --- |
| Average Age (in years) ±St. Dev |  | 49±16 | 49±16 | 48±17 | 48±17 | 48±17 | 47±17 | 47±17 | 46±17 |
| Percent Female |  | 24292 (82.5%) | 31241 (82.6%) | 36816 (82.5%) | 42812 (82.8%) | 45378 (82.9%) | 50669 (82.8%) | 53858 (82.7%) | 57523 (82.5%) |
| Race |  |  |  |  |  |  |  |  |  |
|  | American Indian or Alaska Native | 338 (1.1%) | 458 (1.2%) | 563 (1.3%) | 637 (1.2%) | 742 (1.4%) | 796 (1.3%) | 835 (1.3%) | 911 (1.3%) |
|  | Asian | 438 (1.5%) | 587 (1.6%) | 709 (1.6%) | 841 (1.6%) | 858 (1.6%) | 1039 (1.7%) | 1103 (1.7%) | 1204 (1.7%) |
|  | Black or African American | 3804 (12.9%) | 4742 (12.5%) | 5636 (12.6%) | 6764 (13.1%) | 7244 (13.2%) | 8180 (13.4%) | 8781 (13.5%) | 9728 (14%) |
|  | Native Hawaiian or Other Pacific Islander | 119 (0.4%) | 160 (0.4%) | 185 (0.4%) | 204 (0.4%) | 219 (0.4%) | 249 (0.4%) | 260 (0.4%) | 276 (0.4%) |
|  | Other | 3217 (10.9%) | 4262 (11.3%) | 5072 (11.4%) | 6014 (11.6%) | 6739 (12.3%) | 7921 (12.9%) | 8727 (13.4%) | 9444 (13.5%) |
|  | White | 24369 (82.8%) | 31401 (83.1%) | 36927 (82.8%) | 42631 (82.4%) | 45011 (82.3%) | 50179 (82%) | 53370 (81.9%) | 56709 (81.3%) |
| Ethnicity |  |  |  |  |  |  |  |  |  |
|  | Hispanic or Latino | 1871 (6.4%) | 2430 (6.4%) | 3017 (6.8%) | 3485 (6.7%) | 3824 (7%) | 4478 (7.3%) | 4898 (7.5%) | 5323 (7.6%) |
|  | Not Hispanic or Latino | 26258 (89.2%) | 33684 (89.1%) | 39632 (88.8%) | 45884 (88.7%) | 48325 (88.3%) | 53822 (88%) | 57241 (87.9%) | 61059 (87.6%) |
|  | None of the above | 1300 (4.4%) | 1691 (4.5%) | 1969 (4.4%) | 2338 (4.5%) | 2568 (4.7%) | 2894 (4.7%) | 3009 (4.6%) | 3341 (4.8%) |
| Medications |  |  |  |  |  |  |  |  |  |
| Any Acute Medication |  | 13226 | 17814 | 21547 | 25386 | 28724 | 34250 | 37636 | 41198 |
| Sumatriptan |  | 8535 (64.5%) | 11197 (62.9%) | 13315 (61.8%) | 15282 (60.2%) | 15873 (55.3%) | 17548 (51.2%) | 18167 (48.3%) | 18969 (46%) |
| Rizatriptan |  | 4028 (30.5%) | 5833 (32.7%) | 7359 (34.2%) | 9190 (36.2%) | 10121 (35.2%) | 11801 (34.5%) | 12861 (34.2%) | 14015 (34%) |
| Eletriptan |  | 1334 (10.1%) | 1644 (9.2%) | 1859 (8.6%) | 2211 (8.7%) | 2284 (8%) | 2495 (7.3%) | 2563 (6.8%) | 2605 (6.3%) |
| Almotriptan |  | 186 (1.4%) | 257 (1.4%) | 312 (1.4%) | 419 (1.7%) | 376 (1.3%) | 386 (1.1%) | 369 (1%) | 358 (0.9%) |
| Zolmitriptan |  | 1209 (9.1%) | 1699 (9.5%) | 1882 (8.7%) | 2165 (8.5%) | 2100 (7.3%) | 2052 (6%) | 2011 (5.3%) | 2029 (4.9%) |
| Frovatriptan |  | 355 (2.7%) | 471 (2.6%) | 557 (2.6%) | 606 (2.4%) | 589 (2.1%) | 575 (1.7%) | 559 (1.5%) | 546 (1.3%) |
| Naratriptan |  | 1294 (9.8%) | 1777 (10%) | 2112 (9.8%) | 2561 (10.1%) | 2610 (9.1%) | 3013 (8.8%) | 3133 (8.3%) | 3086 (7.5%) |
| Rimegepant |  | 0 (0%) | 0 (0%) | 0 (0%) | 0 (0%) | 2424 (8.4%) | 6514 (19%) | 8787 (23.3%) | 10714 (26%) |
| Ubrogepant |  | 0 (0%) | 0 (0%) | 0 (0%) | 0 (0%) | 3198 (11.1%) | 5414 (15.8%) | 6670 (17.7%) | 8072 (19.6%) |
| Lasmiditan |  | 0 (0%) | 0 (0%) | 0 (0%) | 0 (0%) | 382 (1.3%) | 675 (2%) | 733 (1.9%) | 766 (1.9%) |
